# Aggressive Mesenchymal Chondrosarcoma of the Rib With Early Skeletal Metastases: A Diagnostic Challenge

**DOI:** 10.7759/cureus.73718

**Published:** 2024-11-15

**Authors:** Francisco Gonçalves, Ricardo L Silva Veiga, Hugo Ventura

**Affiliations:** 1 Internal Medicine, Centro Hospitalar Tondela Viseu, Viseu, PRT; 2 Internal Medicine, Hospital de São Teotónio, Viseu, PRT

**Keywords:** aggressive malignancy, biphasic histopathology, chondrosarcoma, diagnostic and therapeutic challenge, mesenchymal chondrosarcoma, multidisciplinary management

## Abstract

Mesenchymal chondrosarcoma (MCS) is a rare, aggressive subtype of chondrosarcoma characterized by biphasic histology, often misdiagnosed due to its rarity and histological resemblance to other small round cell tumors. It predominantly affects adolescents and young adults.

We report a 27-year-old male presenting with a progressively enlarging, painless mass in the right inframammary region, initially attributed to muscular strain. Over one month, the mass rapidly increased in size, accompanied by mild pain exacerbated by movement. Physical examination revealed a firm, non-tender mass approximately 10 x 5 cm in diameter between the sixth and seventh ribs.

Contrast-enhanced CT revealed a large expansile lytic lesion of the right sixth rib (10 × 5.5 cm) with cortical destruction, pathological fracture and soft tissue invasion into surrounding musculature. Core needle biopsy revealed a biphasic tumor comprising undifferentiated mesenchymal cells and islands of well-differentiated hyaline cartilage. Immunohistochemical staining showed positivity for vimentin, CD99 and S100 protein with a high Ki-67 proliferation index (30-40%). These findings were consistent with MSC. 18-Fluorodeoxyglucose-positron emission tomography (FDG-PET) scan detected widespread skeletal metastases involving the spine, humerus, scapula, ribs, pelvis and femur. A multidisciplinary team initiated neoadjuvant anthracycline-based chemotherapy to reduce tumor burden and manage systemic disease, with plans for potential surgical intervention upon reassessment.

Given the typically poor prognosis associated with MCS, it should be considered in the differential diagnosis of bone tumors in young adults presenting with atypical musculoskeletal masses. Prompt diagnostic workup, including advanced imaging and comprehensive histopathological and immunohistochemical analysis, is essential for accurate diagnosis. A multidisciplinary approach to management is imperative to address the aggressive nature of MCS. Early detection and intervention remain key factors in enhancing survival rates and quality of life for patients afflicted with this formidable malignancy.

## Introduction

Chondrosarcomas (CCS) constitute a heterogeneous group of malignant bone neoplasms characterized by the production of a cartilaginous matrix [1]. They are the third most common primary malignancy of bone, accounting for approximately 20-27% of all primary bone sarcomas [2,3]. Among these, mesenchymal chondrosarcoma (MCS) is an exceedingly rare and aggressive subtype, accounting for approximately 1-10% of all CCS. According to data from the Surveillance, Epidemiology, and End Results (SEER) database between 1973 and 2013, only 226 cases of MCS were reported [4,5].

Histologically, MCS is distinguished by a biphasic pattern comprising undifferentiated small round or spindle-shaped mesenchymal cells interspersed with islands of well-differentiated hyaline cartilage [6]. This unique morphology can pose diagnostic challenges, often leading to misdiagnosis as other small round cell tumors [1]. Immunohistochemical detection of the corresponding biomarkers can help distinguish MCS from other tumors of similar appearance. MCS stains positive for S-100 and SOX9, like CCS [7]; however, it tends to be CD99, ezrin, and NKX2.2 positive, in contrast to CCS [8,9].

MCS can arise in both skeletal and extraskeletal sites. Skeletal lesions frequently involve the axial skeleton, with a predilection for craniofacial bones, particularly the jaws, ribs, pelvis, and vertebrae. The tumor may also present in the appendicular skeleton and can involve multiple sites simultaneously [10]. Extraskeletal manifestations account for approximately one-third of cases, commonly occurring in soft tissues and within the central nervous system, where the meninges are a favored location [11].

Clinically, MCS typically affects adolescents and young adults, with a peak incidence in the second and third decades of life, contrasting with conventional CCS, which predominantly affects individuals over 50 years of age [12]. MCS exhibits a more aggressive clinical course than CCS, characterized by a high propensity for local recurrence and distant metastasis, particularly to the lungs and bones. The five-year overall survival rate for patients with MCS is approximately 55%, with metastatic disease present at diagnosis in nearly 20% of cases [1,13].

The initial presentation of MCS is often insidious, with nonspecific symptoms related to the mass effect of the tumor, such as pain or a palpable mass. Due to the rarity of the disease and the nonspecific nature of early symptoms, diagnosis is frequently delayed, and patients may initially be referred to non-oncologic specialties [14].

MCS treatment is limited due to its high malignancy and metastatic potential, so new potential therapeutic targets are needed [15]. The treatment of patients with MCS depends on the primary site, the extent of the disease, the presence of distant metastases at diagnosis, and the medical condition of the patient. Early recognition and accurate diagnosis are critical for optimizing treatment outcomes, which typically involve a combination of surgical resection, chemotherapy and radiation therapy. We report a case of a young male patient presenting with a rapidly enlarging chest wall mass, ultimately diagnosed as MCS with extensive skeletal metastases, highlighting the aggressive nature of this rare malignancy and the challenges in its diagnosis and management.

## Case presentation

A 27-year-old male amateur boxer and kickboxer presented to the emergency department with a progressively enlarging mass in the right inframammary region. The patient reported that six months prior, he noticed a small, painless lump in the right costal area, which he initially attributed to muscular strain from training. Over the past month, he observed a rapid increase in the size of the mass, coinciding with mild, persistent pain localized to the right costal margin. The pain was exacerbated by certain movements but was manageable with over-the-counter analgesics. He denied any history of trauma, including during boxing or kickboxing activities, and could not recall any specific incident that might have precipitated the mass's onset.

The patient had no significant past medical history and was not on any regular medications. He reported taking creatine monohydrate and vitamin D supplements but denied the use of anabolic steroids or other performance-enhancing substances. There was no history of weight loss, night sweats, fever, dyspnea, or fatigue.

On physical examination, the patient was well-appearing and hemodynamically stable and afebrile. Inspection and palpation revealed a firm, non-tender, poorly mobile mass measuring approximately 10 x 5 cm in the right inframammary region, situated between the sixth and seventh ribs. The overlying skin was intact, without erythema, warmth, or visible pulsation. There was no evidence of superficial venous engorgement or lymphadenopathy in the cervical, axillary, or inguinal regions. Cardiovascular examination revealed a regular rhythm without murmurs or extra heart sounds. Pulmonary auscultation demonstrated clear bilateral breath sounds without wheezes, rales, or rhonchi. The abdomen was soft and non-tender, with no organomegaly or signs of peritoneal irritation. Neurological examination was unremarkable.

Initial laboratory studies, including complete blood count and metabolic panel, were within normal limits except for a mildly elevated alkaline phosphatase. Liver function tests, renal function tests, and inflammatory markers were unremarkable (Table 1).

**Table 1:** Summary of laboratory results CRP: C-reactive protein

Test	Result	Reference Range
Leukocytes	9.96 x 10^9/L	4.0-11.0 x 10^9/L
Neutrophils	76.2% 7.6 x 10^9/L	40-80% 2.0-7.5 x 10^9/L
Lymphocytes	17.6% 1.8 x 10^9/L	20-45% 1.0-4.5 x 10^9/L
Hemoglobin	16.4 g/dL	13.5-17.5 g/dL
Platelets	332.0 x 10^9/L	150-450 x 10^9/L
Urea	37 mg/dL	15-40 mg/dL
Creatinine	1.0 mg/dL	0.6-1.2 mg/dL
Sodium	137 mEq/L	135-145 mEq/L
Potassium	4.1 mEq/L	3.5-5.0 mEq/L
Chloride	101.9 mEq/L	98-106 mEq/L
Calcium	10.4 mg/dL	8.5-10.5 mg/dL
Phosphorus	4.0 mg/dL	2.5-4.5 mg/dL
Total Proteins	7.0 g/dL	6.0-8.3 g/dL
Albumin	4.8 g/dL	3.5-5.0 g/dL
Alkaline Phosphatase	150 Ul/L	45-117 Ul/L
Gamma-glutamyltransferase	15.8 UI/L	9-48 UI/L
Alanine Aminotransferase	27 Ul/L	7-56 Ul/L
Aspartate Aminotransferase	31 Ul/L	5-40 Ul/L
Lactate Dehydrogenase	236 Ul/L	140-280 Ul/L
Total Creatine Phosphokinase	89 UI/L	30-200 UI/L
Total Bilirubin	1.0 mg/dL	0.1-1.2 mg/dL
Direct Bilirubin	0.24 mg/dL	0.0-0.3 mg/dL
High-sensitivity CRP	0.02 mg/dL	<0.5 mg/dL
Myoglobin	11.00 ng/mL	0.0-110.0 ng/mL

Serologic tests for HIV, hepatitis B virus (HBV), and hepatitis C virus (HCV) were negative. Imaging studies were reviewed. A chest radiograph obtained by the primary care physician showed an opacity in the right lower hemithorax, appearing to involve the anterior arch of the seventh rib, with evidence of cortical disruption, extending into the adjacent soft tissues. A diagnostic ultrasound of the mass revealed a solid, heterogeneous lesion measuring approximately 5 × 6 cm with irregular borders and internal vascularity.

**Figure 1:**
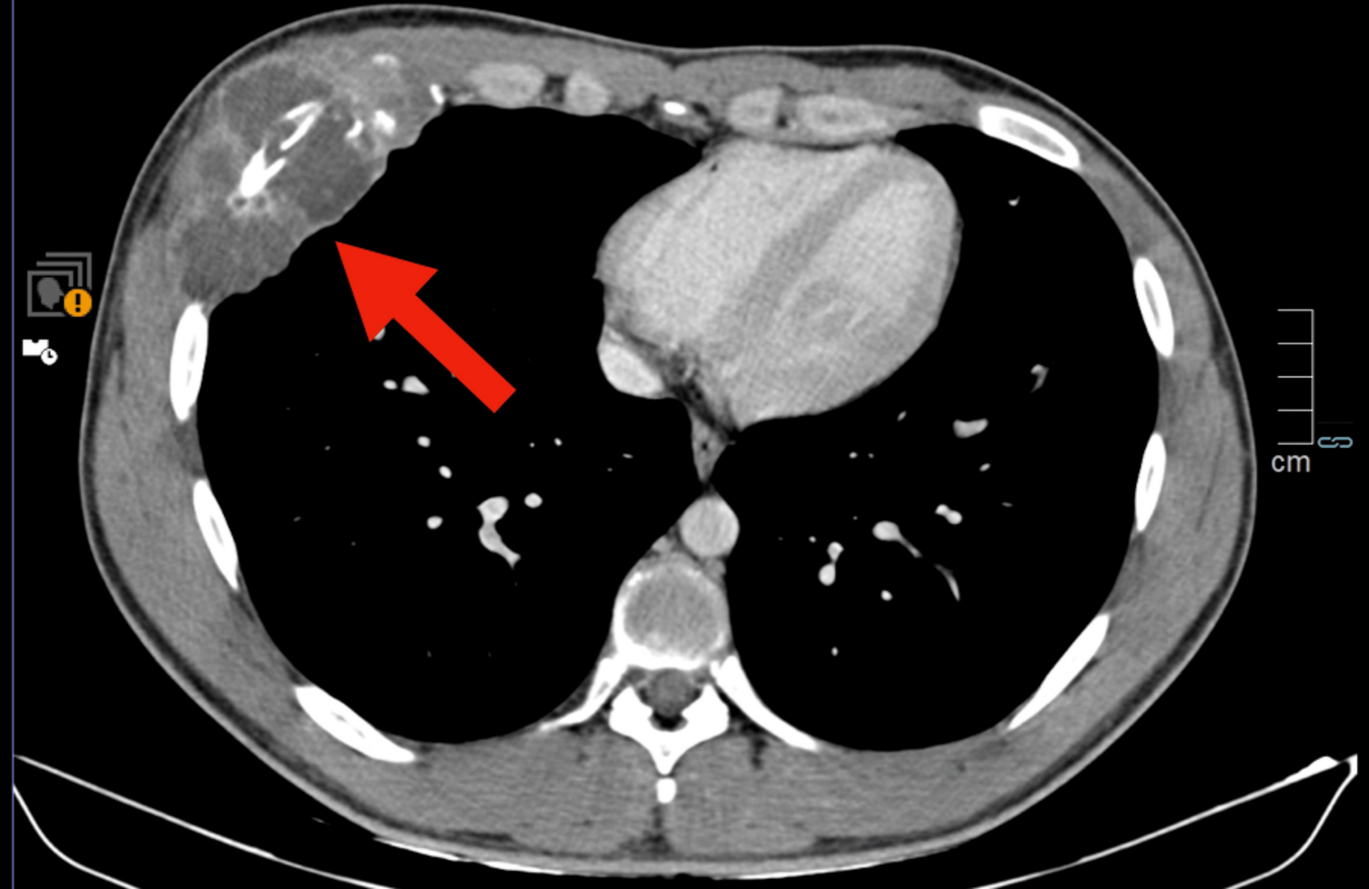
Axial contrast-enhanced CT scan of the thorax Voluminous soft-tissue lytic neoformation infiltrating the anterior aspect of the right sixth rib. This lesion shows heterogeneous contrast enhancement and some peripheral calcifications (red arrow).

**Figure 2:**
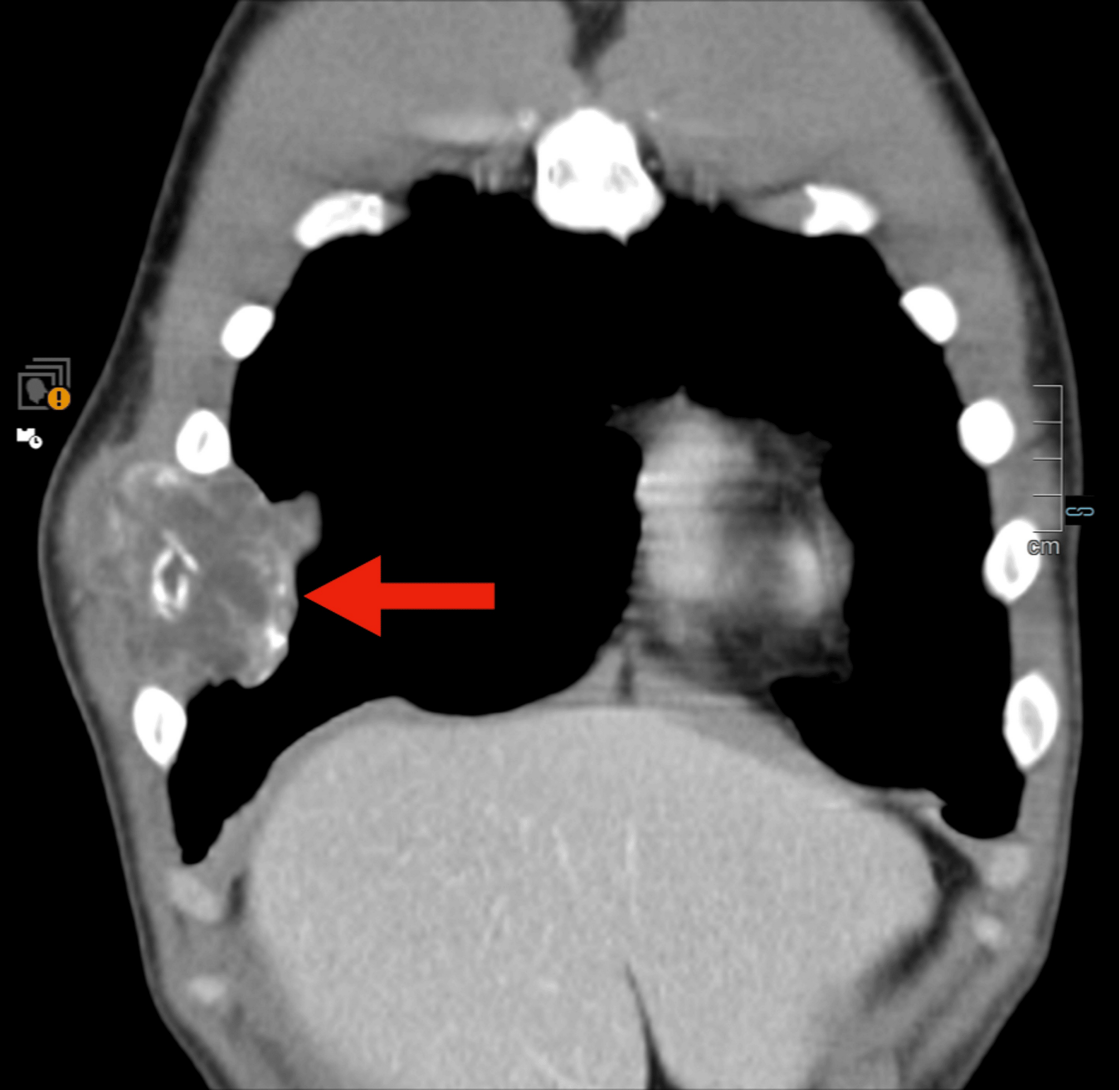
Coronal contrast-enhanced CT scan of the thorax Voluminous soft-tissue lytic neoformation infiltrating the anterior aspect of the right sixth rib. This lesion shows heterogeneous contrast enhancement and some peripheral calcifications (red arrow).

**Figure 3:**
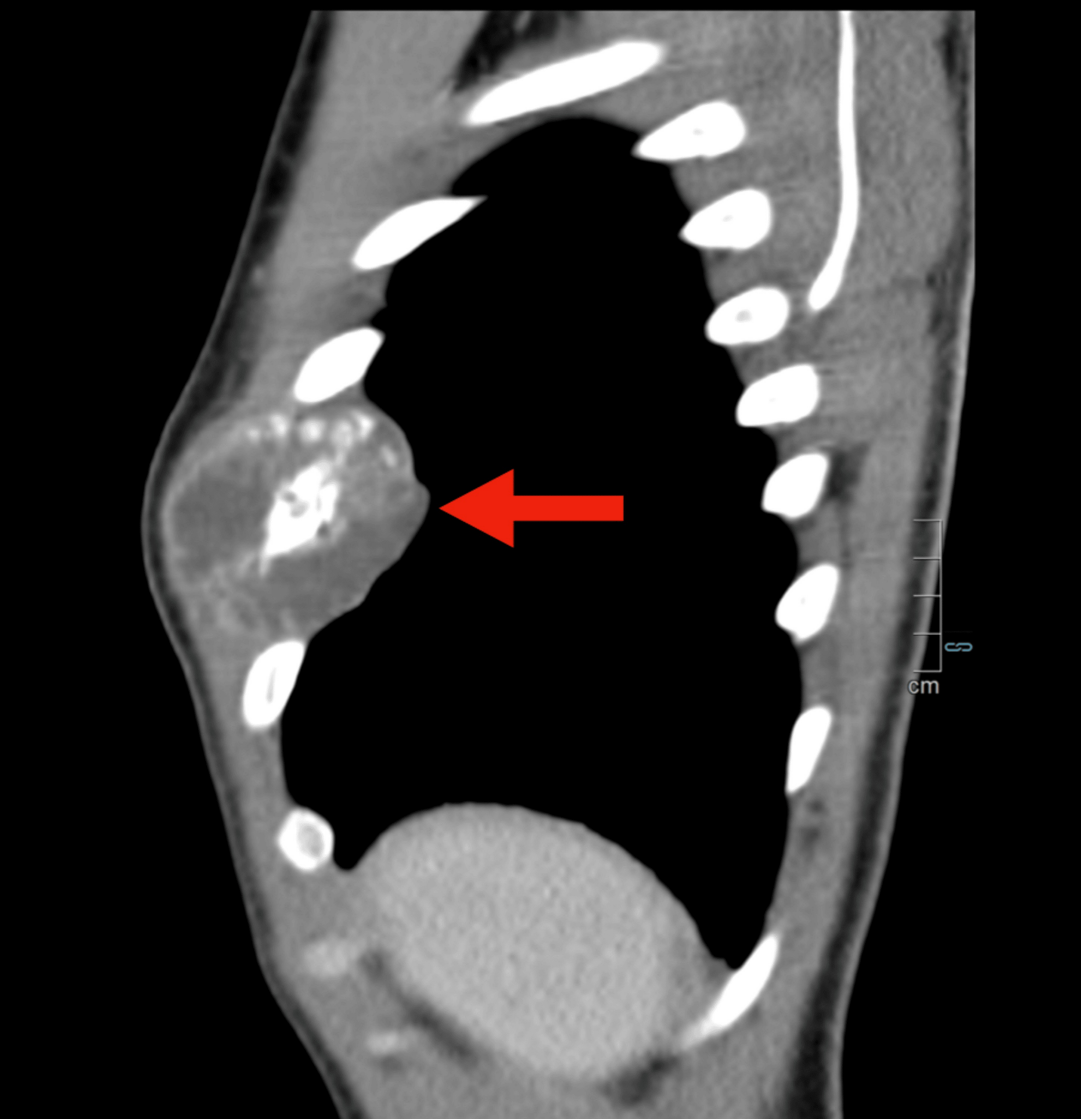
Sagittal contrast-enhanced CT scan of the thorax Voluminous soft-tissue lytic neoformation infiltrating the anterior aspect of the right sixth rib. This lesion shows heterogeneous contrast enhancement and some peripheral calcifications (red arrow).

Given the concerning imaging findings, a contrast-enhanced computed tomography (CT) scan of the thorax, abdomen, and pelvis was performed. The CT scan demonstrated a large expansile lytic lesion involving the anterior aspect of the right sixth rib, measuring approximately 10 × 5.5 cm (Figures 1-3).

There was cortical destruction with a pathological fracture of the rib and an associated soft tissue mass extending into the surrounding musculature. The lesion exhibited heterogeneous enhancement after contrast administration. No additional osseous lesions or visceral abnormalities were identified on the CT scan.

**Figure 4:**
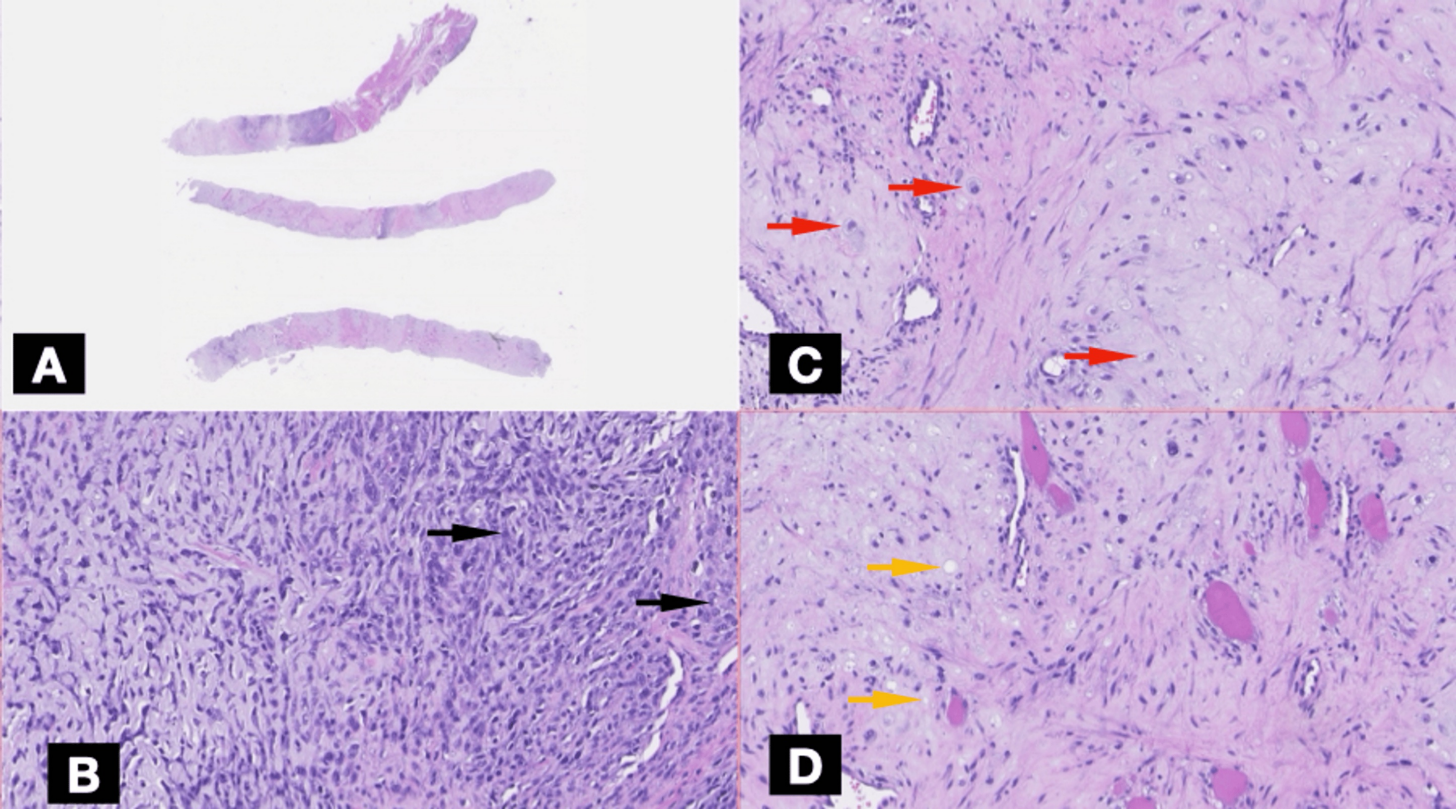
Histopathological features Panel A shows three fragments obtained from a needle-guided biopsy. Panel B shows undifferentiated small round to spindle-shaped mesenchymal cells (black arrows). Panel C shows islands of well-differentiated hyaline cartilage with chondroblasts (red arrows). Panel D shows islands of well-differentiated hyaline cartilage with lacunar spaces (yellow arrows).

**Figure 5:**
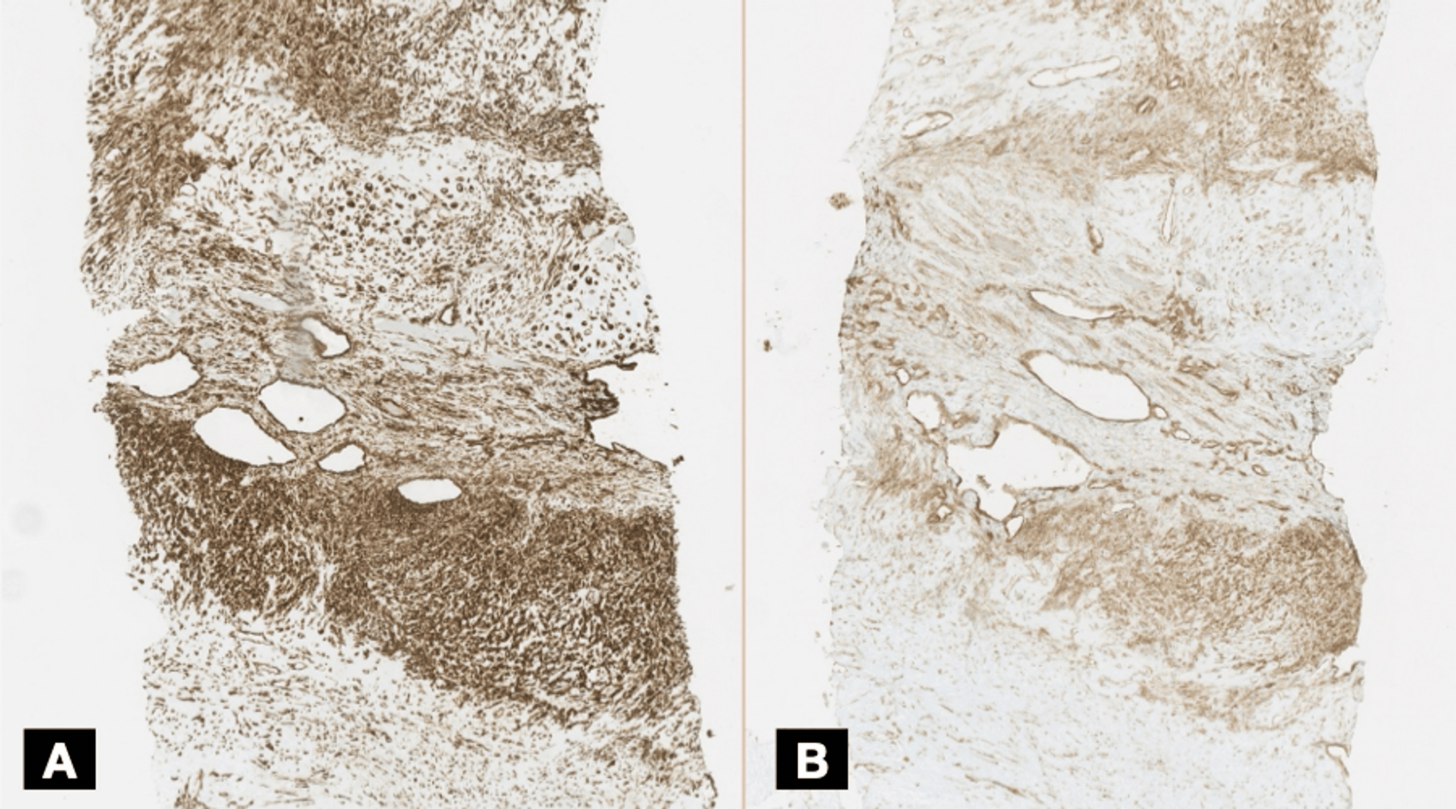
Immunohistochemical analysis of tumor components Panel A shows positive staining for vimentin in the mesenchymal component. Panel B shows positive staining for CD99.

**Figure 6:**
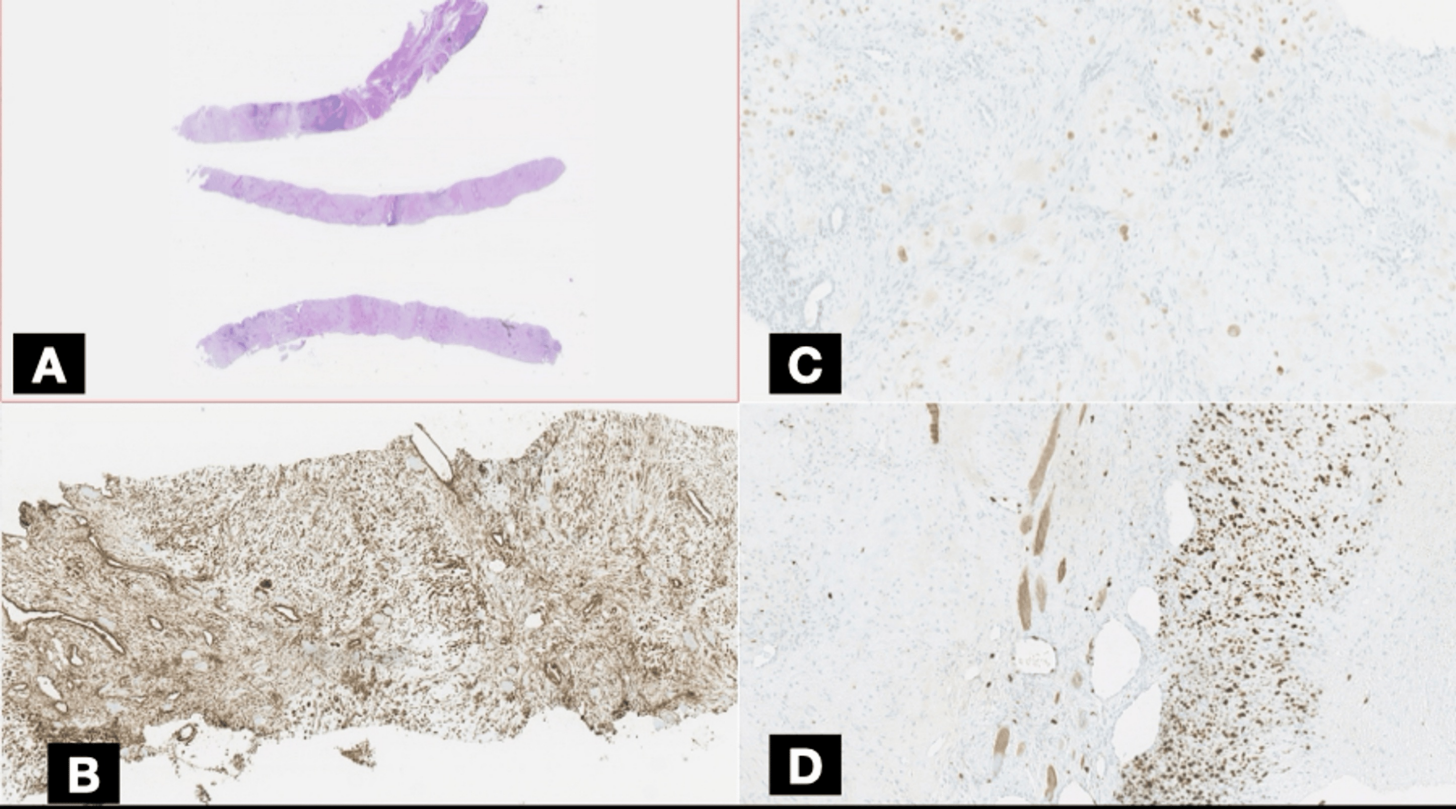
Immunohistochemical analysis of tumor components Panel A shows three fragments obtained from a needle-guided biopsy. Panel B shows positive staining for vimentin in the mesenchymal component. Panel C shows S100 positivity in cartilaginous areas. Panel D shows an elevated Ki-67 proliferation index.

An ultrasound-guided core needle biopsy of the rib lesion was performed without complications. Histopathological examination revealed a biphasic tumor composed of sheets of undifferentiated small round to spindle-shaped mesenchymal cells with hyperchromatic nuclei and scant cytoplasm, interspersed with islands of well-differentiated hyaline cartilage (Figure 4). Immunohistochemical staining showed diffuse positivity for vimentin and CD99 in the mesenchymal component and S100 protein was positive in the cartilaginous areas (Figures 5, 6). The tumor cells were negative for pancytokeratin (AE1/AE3), epithelial membrane antigen (EMA), desmin, smooth muscle actin (SMA), calretinin, and CD117. The Ki-67 proliferation index was elevated at 30-40% in the small cell component, indicating high mitotic activity (Figure 5). These findings were consistent with a diagnosis of MCS.

**Figure 7:**
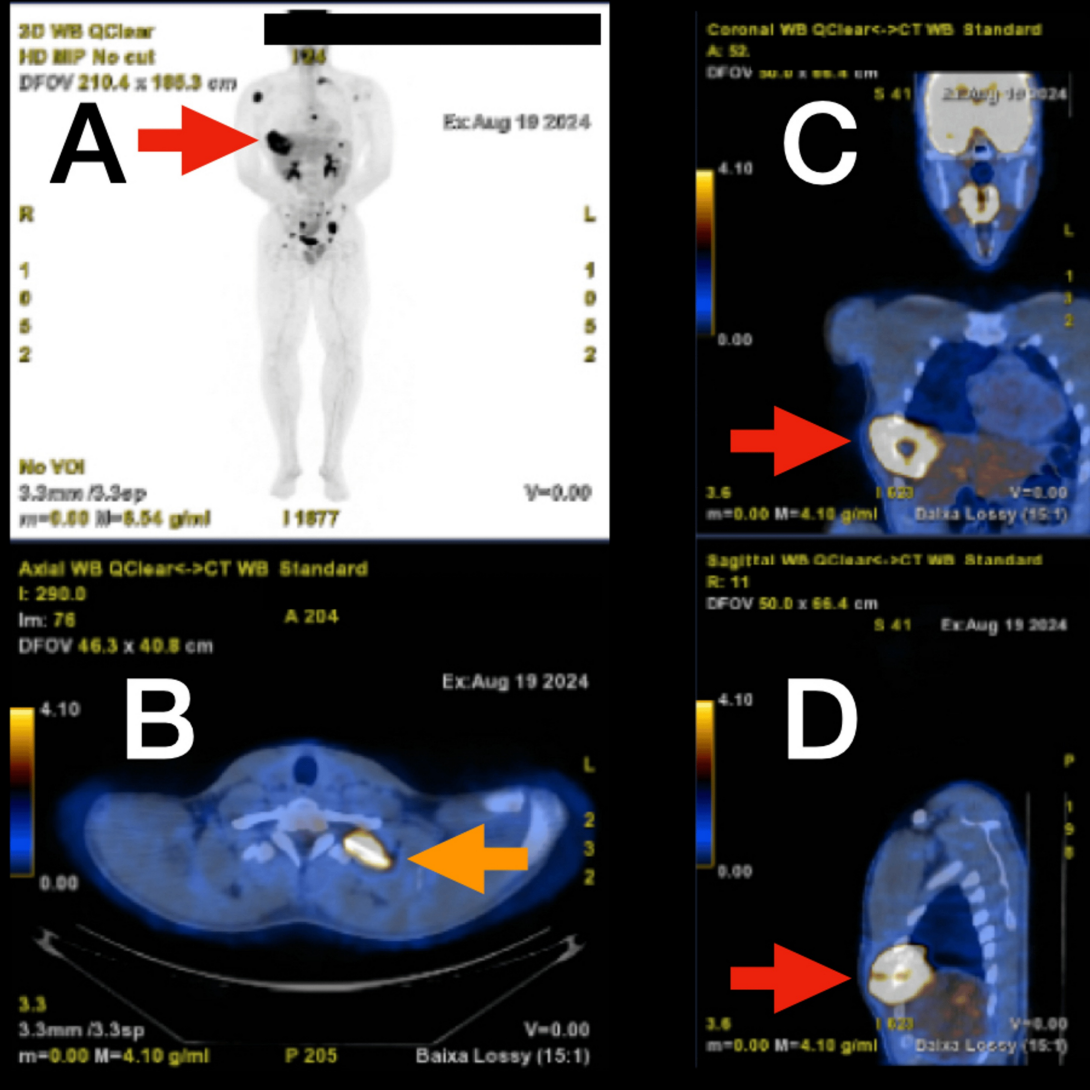
Whole-body PET scan with FDG Panel A shows the primary lesion surrounded by widespread skeletal metastases (red arrow). Panel B shows intense FDG uptake in the secondary lesion on the left second rib (orange arrow). Panels C and D show intense FDG uptake in the primary rib lesion (red arrow). PET: positron emission tomography; FDG: fluorodeoxyglucose

The patient was promptly referred to a tertiary sarcoma center for further evaluation and management. A whole-body positron emission tomography (PET) scan with fluorodeoxyglucose (FDG) was performed, revealing intense FDG uptake in the primary rib lesion. Additionally, the PET scan demonstrated widespread skeletal metastases with increased metabolic activity involving the thoracic and lumbar spine, right humerus, left scapula, left second rib, pelvic bones, and right femur, and no visceral metastases were identified (Figure 7).

Given the extensive metastatic disease, the patient was evaluated by a multidisciplinary team comprising internal medicine, oncology, radiology and thoracic surgery specialists. The consensus was to initiate neoadjuvant chemotherapy with an anthracycline-based regimen to reduce tumor burden and address systemic disease, followed by reassessment for potential surgical intervention. The patient was counseled regarding his diagnosis, prognosis, and treatment options, and he provided informed consent to proceed with therapy.

## Discussion

MCS is an exceptionally rare and aggressive malignancy, accounting for approximately 1-10% of all CCS and presenting significant diagnostic and therapeutic challenges [4,5]. This case exemplifies the prototypical features of MCS, including its occurrence in a young adult, as it typically affects individuals in their second and third decades of life, contrasting with conventional CCS that generally affects those over 50 years old [12], axial skeletal involvement, aggressive behavior with early metastasis [10], and the characteristic biphasic histopathological pattern.

The patient's initial presentation with a painless, slowly enlarging chest wall mass aligns with the often-insidious onset of MCS. The absence of systemic symptoms such as weight loss, fever, or night sweats contributed to a delay in seeking medical attention, a common issue among young, otherwise healthy individuals. This delay is characteristic of MCS, as the diagnosis is frequently postponed due to nonspecific signs and symptoms and referrals to non-oncologic specialties [14]. The patient's daily practice of martial arts activities, including boxing and kickboxing, led him to underestimate the growing mass, attributing it to strenuous physical exercise. The rapid growth of the mass over the past month, accompanied by mild pain exacerbated by movement, was the decisive factor that prompted the patient to seek medical help. These changes underscore the aggressive nature of the tumor.

Radiologically, the expansile lytic lesion with cortical destruction and a soft tissue component is characteristic of malignant bone tumors. However, histopathological examination was crucial for definitive diagnosis, given the rarity of this entity. The biphasic pattern of undifferentiated small round to spindle-shaped mesenchymal cells interspersed with islands of well-differentiated hyaline cartilage is characteristic of MCS [6]. Immunohistochemistry played a pivotal role in differentiating MCS from other small round-cell tumors. The mesenchymal component's positivity for vimentin and CD99, along with S100 protein expression in the cartilaginous areas, supported the diagnosis [7-9]. The high Ki-67 proliferation index indicated a high-grade tumor with aggressive potential. The detection of widespread skeletal metastases at the time of diagnosis is indicative of the aggressive behavior of MCS. Studies have shown that MCS has a high propensity for early metastasis, with nearly 20% of cases presenting with metastatic disease at the time of diagnosis [1], commonly involving the lungs and bones.

Management of MCS poses significant challenges due to its rarity and aggressive nature. There is no standardized treatment protocol, but a multimodal approach is generally advocated. The management of patients presenting with distant metastases is individualized. Those who are young with limited distant metastases may be considered for chemotherapy combined with aggressive local management with surgery and/or radiotherapy. Patients who are elderly, infirm, and/or with extensive disease may best be managed with palliative intent [16]. In this case, the multidisciplinary team's decision to initiate neoadjuvant chemotherapy aimed to reduce tumor burden and address systemic disease before considering surgical options. Close monitoring of the patient's response to chemotherapy will be essential in guiding further management decisions.

Prognostically, MCS is associated with a poorer outcome compared to conventional CCS, with a five-year survival rate of approximately 55% [13]. Metastatic disease at diagnosis is the primary prognostic factor for reduced survival, however, other factors include tumor size, the axial location of the primary tumor, and age [4,15]. Therefore, the patient's extensive metastatic disease at presentation indicates a guarded prognosis. Prompt identification and proactive treatment play a crucial role in enhancing patient outcomes.

This case highlights the importance of considering MCS in the differential diagnosis of bone tumors in young adults, especially when masses are described as painless lumps involving the axial skeleton. It underscores the need for a high index of suspicion and prompt diagnostic workup, including advanced imaging and tissue biopsy with comprehensive histopathological and immunohistochemical analysis. Only through timely diagnosis can we structure the optimal therapeutic plan with a multidisciplinary team to confront such a rare and aggressive neoplasm from the outset.

## Conclusions

This case underscores the aggressive nature and diagnostic challenges of MCS, a rare malignancy that predominantly affects young adults. The patient's initial presentation with a painless, slowly enlarging chest wall mass without systemic symptoms led to a delay in seeking medical attention, a common occurrence in MCS due to its insidious onset and nonspecific clinical features. The rapid progression and early skeletal metastasis highlight the critical importance of maintaining a high index of suspicion for malignant neoplasms in young patients presenting with unusual musculoskeletal masses.

Early recognition and accurate diagnosis of MCS are essential for optimizing treatment outcomes, given its poor prognosis and lack of standardized therapeutic protocols. This case emphasizes the need for prompt diagnostic workup, including advanced imaging and thorough histopathological and immunohistochemical analysis, to differentiate MCS from other small round cell tumors. A multidisciplinary approach is imperative for managing such aggressive neoplasms, allowing for the integration of systemic chemotherapy and consideration of surgical intervention when feasible.
